# Perceptions and experiences of missed nursing care among junior nurses: a qualitative study using reflexive thematic analysis

**DOI:** 10.3389/frhs.2026.1853426

**Published:** 2026-07-20

**Authors:** Rongrong Zhou, Xiaojuan Liu, Bihua shen, Shuirong Zhan, Shu Zhou, Yanjun Mao

**Affiliations:** 1 Department of Nursing, Shanghai Yangzhi Rehabilitation Hospital (Shanghai Sunshine Rehabilitation Hospital), Shanghai, China; 2 Department of Nursing, Jing'an District Traditional Chinese Medicine Hospital, Shanghai, China

**Keywords:** missed nursing care, junior nurses, patient safety, qualitative research, reflexive thematic analysis, nursing workforce

## Abstract

**Background:**

Missed nursing care is an important indicator of healthcare quality and patient safety. Junior nurses undergoing the transition from student to professional practitioner are particularly vulnerable to missed nursing care due to limited clinical experience, insufficient professional competence, and challenges in adapting to complex clinical environments. However, the multidimensional factors contributing to missed nursing care among junior nurses remain insufficiently understood.

**Methods:**

A qualitative descriptive study was conducted involving 12 junior nurses with ≤3 years of clinical experience. Participants were recruited through purposive sampling. Data were collected using semi-structured, in-depth interviews and analyzed using Braun and Clarke's six-phase thematic analysis approach. This study aimed to explore junior nurses’ perceptions and experiences of missed nursing care and to identify the underlying factors contributing to its occurrence in clinical practice.

**Results:**

Three overarching themes and eight subthemes emerged from the analysis. (1) Individual factors, including insufficient knowledge and clinical skills, psychological stress and challenges in professional role transition, and weakened professional responsibility; (2) Organizational factors, including inadequate staffing and excessive workload, deficiencies in quality management and supervisory support, and limited career development opportunities and incentive mechanisms; and (3) External environmental factors, including challenges associated with healthcare system transformation and increasing expectations from patients and family members. Participants reported that missed nursing care often resulted from the interaction of personal limitations, organizational constraints, and external pressures.

**Conclusions:**

Junior nurses' experiences of missed nursing care are shaped by multidimensional influences at the individual, organizational, and external levels. Addressing missed nursing care requires comprehensive and multilevel interventions, including competency-based education, strengthened psychological and transition support, optimized nursing management systems, and enhanced institutional and social support. Such efforts may help reduce missed nursing care and improve the quality and safety of nursing services.

## 1 Background

The global health agenda of the World Health Organization (WHO) and the 2030 Agenda for Sustainable Development have established universal health coverage (UHC) as a fundamental principle of global healthcare systems (1, 2). Universal health coverage emphasizes not only equitable access to healthcare services but also the provision of safe, high-quality, and person-centered care that addresses patients' comprehensive needs (3). However, amid the ongoing transformation toward person-centered healthcare, missed nursing care has increasingly emerged as a major challenge to care quality and patient safety.

Missed nursing care refers to any required nursing care that is delayed, partially completed, or omitted, including essential nursing activities such as patient repositioning, oral care, medication monitoring, clinical observation, and health education (4, 5). The concept was first introduced by Beatrice J. Kalisch in 2006 and defined as “any aspect of required patient care that is omitted or delayed” during the delivery of nursing care. Since then, a growing body of international evidence has demonstrated that missed nursing care is widespread across healthcare systems and is closely associated with adverse patient outcomes, including falls, pressure injuries, medication errors, prolonged hospitalization, and reduced patient satisfaction (6–9). At the workforce level, missed nursing care has also been linked to increased nurse burnout, reduced job satisfaction, emotional exhaustion, and higher turnover intention (10, 11).

Fundamentally, missed nursing care reflects a critical paradox in clinical practice: despite the existence of professional standards, clinical protocols, and institutional guidelines, nurses may still be unable to complete all necessary nursing tasks because of competing demands and multiple contextual constraints. Consequently, missed nursing care has become an increasingly important focus of nursing workforce research and healthcare quality improvement initiatives.

Existing literature has predominantly examined the prevalence, typologies, and organizational determinants of missed nursing care, particularly factors such as nurse staffing levels, workload, time pressure, and resource availability (8, 12). By comparison, relatively limited attention has been paid to individual-level determinants, including nurses' clinical competence, psychological status, professional responsibility, and adaptive capacity (13). Moreover, although some studies have explored workplace-related influences, the multidimensional and interactive mechanisms underlying missed nursing care remain insufficiently understood. In particular, nurses' subjective perceptions and experiences of missed nursing care, as well as the complex interplay among individual, organizational, and external environmental factors, have received limited scholarly attention.

This knowledge gap is especially important in the Chinese healthcare context, where increasing service demand, relatively constrained nursing resources, and ongoing healthcare reforms may intensify pressures on frontline nurses. Simultaneously, the continued promotion of person-centered care models and high-quality healthcare services has expanded expectations regarding nursing performance, further increasing the complexity of nursing practice. Under these circumstances, junior nurses—who are undergoing the transition from student to independent practitioner—may be particularly vulnerable to missed nursing care because of limited experience, developing clinical competence, and challenges in professional adaptation.

Reducing missed nursing care is essential to advancing person-centered nursing practice and improving healthcare quality and patient safety (14, 15). A better understanding of the factors contributing to missed nursing care may help healthcare managers optimize staffing allocation, improve workflow efficiency, strengthen supervisory support, and enhance nursing competency development (16, 17). Furthermore, increasing nurses' awareness of missed nursing care may encourage reflective practice, strengthen professional accountability, and improve the consistency and comprehensiveness of nursing care. Such efforts may not only improve patient outcomes but also promote nurses' professional fulfillment and long-term career development (18, 19).

Therefore, using a Qualitative Study approach, this study aims to explore junior nurses' perceptions and experiences of missed nursing care, identify the multidimensional factors contributing to its occurrence, and examine potential strategies for prevention and improvement. By providing an in-depth understanding of missed nursing care from the perspective of junior nurses, this study seeks to inform the development of targeted interventions and support the delivery of safer, higher-quality nursing care.

## 2 Methods

### 2.1 Study design

A qualitative study was conducted to explore junior nurses' perceptions and experiences of missed nursing care incidents. This design was considered appropriate for obtaining an in-depth understanding of participants' subjective experiences and perceptions within real-world clinical contexts. Purposive sampling with maximum variation was used to recruit junior nurses with diverse demographic and professional backgrounds. To ensure variation in perspectives, participants were selected to reflect differences in gender, educational level (diploma, bachelor's, and master's degree), department, and years of clinical experience (≤3 years). An initial sample of 12 junior nurses who expressed a willingness to share their experiences was planned.

The sample size was guided by the concept of information power rather than numerical saturation. Participants were purposively recruited because they had direct experience with missed nursing care during their early clinical practice. Semi-structured interviews lasted approximately 45–60 min, generating rich and detailed narratives. Recruitment ceased after 12 interviews when the research team determined that the dataset provided sufficient information power, diversity of experiences, and analytical depth to comprehensively address the study aims.

### 2.2 Sampling techniques

A purposive sampling strategy with maximum variation was employed to recruit junior nurses who were able to provide rich and relevant insights into missed nursing care. Participants were intentionally selected from nurses who had expressed willingness to share their experiences, with efforts made to ensure diversity in gender, educational background (diploma, bachelor's, and master's degree), clinical department, and years of clinical experience (≤3 years).

Maximum variation sampling was used to capture a broad range of perspectives and experiences related to missed nursing care among junior nurses. This approach facilitated the collection of in-depth, contextually relevant data and enhanced the comprehensiveness of the findings by incorporating participants from diverse professional and demographic backgrounds.

### 2.3 Data collection tools

We used a semi-structured interview guide to facilitate participant-centred conversations. This approach provided the flexibility to pose follow-up questions and explore participants' responses in greater depth. The interview questions included: 1. —Could you share your experience of missed nursing care during your time as a junior nurse? Can you describe a specific incident that comes to mind? How do you perceive omissions of nursing care? What do you think are the various causes contributing to missed nursing care? What do you think is the impact of missed nursing care on patients? What were your feelings or reflections when facing such an event? How do you perceive the impact of missed nursing care on your overall education and professional development? What kind of support do you think is needed to address missed nursing care? Is there any additional information you would like to share that we have not covered? (Supplementary Material S2).

### 2.4 Data collection procedures

Potential participants who expressed a willingness to share their experiences of missed nursing care and provided contact information were approached by the research team. Before the interviews, participants were informed about the study's purpose, interview procedures, confidentiality measures, and their right to withdraw at any stage without consequences. Written informed consent was obtained from all participants prior to data collection.

Individual face-to-face interviews were conducted in a private and quiet room within the hospital to ensure comfort, confidentiality, and minimal interruption. All interviews were audio-recorded using a digital voice recorder with participants' permission. Field notes were taken during and immediately after the interviews to document non-verbal expressions, emotional responses, and contextual details relevant to participants' experiences (20, 21).

To protect confidentiality, each participant was assigned a unique identification code, and all personal identifiers were removed from transcripts and research materials. Audio recordings, transcripts, and field notes were stored securely and were accessible only to authorized members of the research team. Participants were assured that all information shared during the interviews would remain confidential and be used solely for research purposes.

### 2.5 Data analysis

All interviews were audio-recorded and transcribed verbatim shortly after data collection. Data were analyzed using inductive thematic analysis following Braun and Clarke's six-phase framework: (1) familiarization with the data, (2) generating initial codes, (3) searching for themes, (4) reviewing themes, (5) defining and naming themes, and (6) producing the report (22).

The analytical process followed the principles of reflexive thematic analysis. To improve transparency and provide an audit trail of the analysis, the analytical process, representative coding framework, and coding tree are presented in Supplementary Tables S2, S3.

To enhance analytical rigor and ensure credibility, members of the research team independently reviewed the coding structure, discussed discrepancies, and reached consensus regarding theme development and interpretation. Themes were continuously refined to ensure they accurately reflected participants’ experiences and remained closely grounded in the original data. Representative quotations were selected to illustrate each theme and preserve participants' perspectives.

Data analysis was conducted in the original language (Chinese) to maintain conceptual accuracy and contextual meaning. Quotations included in the manuscript were translated into English by one bilingual member of the research team and independently checked by another team member to ensure linguistic accuracy and consistency of meaning.

### 2.6 Trustworthiness

The trustworthiness of the findings was ensured using Lincoln and Guba's criteria of credibility, dependability, confirmability, and transferability (23).

Credibility was enhanced through close engagement with participants during the interviews, enabling the collection of rich and authentic accounts of their experiences. Semi-structured interviews allowed participants to elaborate on their perceptions in depth, while representative quotations were incorporated to accurately reflect participants’ voices. In addition, peer debriefing among members of the research team facilitated critical discussion and validation of data interpretation.

Transferability was supported by providing detailed descriptions of the study context, participant characteristics, sampling strategy, and research procedures, allowing readers to assess the applicability of the findings to other clinical settings. Maximum variation sampling was also employed to capture diverse perspectives among junior nurses from different educational and professional backgrounds.

Dependability was strengthened through the use of a consistent semi-structured interview guide across all interviews, which were conducted by the same researcher to maintain procedural consistency. An audit trail, including interview transcripts, coding decisions, analytic memos, and theme development processes, was maintained throughout the study to enhance transparency and methodological rigor.

Confirmability was promoted through reflexive discussions within the research team to minimize potential interpretive bias. In addition, quotations translated from Chinese to English were reviewed by a second bilingual researcher to ensure linguistic accuracy and preserve the original meaning of participants’ accounts, thereby supporting the credibility and neutrality of the findings.

### 2.7 Ethical considerations

This study was approved by the Institutional Review Board of Shanghai Jing'an District Hospital of Traditional Chinese Medicine (Approval No. 2024-LLSC-61) and conducted in accordance with relevant ethical principles and institutional requirements for research involving human participants.

Prior to participation, all participants received a detailed explanation of the study's purpose, procedures, potential risks and benefits, confidentiality measures, and their right to withdraw from the study at any time without consequences. Written informed consent was obtained from all participants before data collection.

To ensure confidentiality and protect participants’ privacy, each participant was assigned a unique identification code, and all personal identifying information was removed from interview transcripts and study records. Research data were securely stored and accessible only to authorized members of the research team.

## 3 Findings

### 3.1 Theme 1—individual factors contributing to missed nursing care

This theme illustrates how individual-level factors contributed to missed nursing care among junior nurses. Participants described that insufficient clinical competence, psychological burden during professional transition, and attitudes toward professional responsibility collectively influenced their ability to deliver complete and timely nursing care.

#### 3.1.1 Inadequate knowledge and skills are direct barriers

Nearly all participants described insufficient clinical knowledge and limited technical skills as important factors contributing to missed nursing care. Junior nurses reported difficulties in clinical judgment, emergency response, and task prioritization, particularly when managing complex patient conditions or multiple competing demands. These challenges often resulted in delays or omissions in nursing care.

One participant described uncertainty in recognizing and responding to abnormal clinical signs:

“At that time, the patient had an abnormal heart rate, and I only documented it briefly without notifying the physician promptly. This happened because I was not sufficiently confident in my understanding of the criteria for interpreting abnormal heart rates across different clinical conditions. I was uncertain whether the abnormal change required urgent intervention and was worried that misjudgment might cause unnecessary panic. Therefore, I chose to document and observe first, which ultimately delayed the appropriate response.” (C1)

Another participant highlighted difficulties in coordinating multiple nursing responsibilities during emergencies:

“When emergencies occur and I need to care for multiple patients Simultaneously, I become overwhelmed, and some nursing tasks tend to be overlooked. Especially since I had only recently entered clinical practice, I had not yet developed the ability to coordinate multiple nursing responsibilities at the same time. Faced with overlapping patient conditions and procedural demands, I found it difficult to quickly prioritize tasks.” (C2)

Participants further reported that rapidly changing clinical guidelines, emerging technologies, and newly introduced nursing procedures sometimes exceeded their existing competencies. Under such circumstances, some nursing activities were simplified or delayed, increasing the likelihood of missed nursing care. These findings suggest that limitations in knowledge and technical skills were perceived as important individual-level contributors to missed nursing care among junior nurses.

#### 3.1.2 Psychological stress and challenges in role transition

Participants commonly described experiencing considerable psychological stress during the early stage of professional transition. Heavy workloads, night shifts, unfamiliar clinical responsibilities, and the pressure of adapting to a fast-paced healthcare environment were frequently perceived as sources of stress. Participants reported that psychological fatigue and emotional burden sometimes affected their concentration, clinical judgment, and ability to complete nursing tasks.

One participant described persistent work-related pressure and reduced attentiveness:

“The hospital environment is extremely fast-paced, and there are many night shifts. I am constantly under pressure, and sometimes I forget to double-check information.” (C4)

Another participant emphasized the impact of exhaustion on clinical performance:

“Sometimes I am so exhausted that I cannot think clearly and may even make mistakes in medication dosages.” (C2)

Participants also described the transition from student to registered nurse as particularly challenging. Many reported feeling overwhelmed by the intensity of frontline nursing work and the need to manage multiple competing responsibilities simultaneously.

“The transition from being a student to becoming a nurse happened too quickly. When I first started working, I felt completely overwhelmed by the number of tasks that needed to be handled simultaneously, and I often missed basic nursing procedures.” (C8)

Participants suggested that limited clinical experience and insufficient coping strategies during professional transition made it difficult to prioritize and coordinate nursing tasks effectively, particularly in demanding clinical situations. Under such circumstances, omissions in routine nursing care were perceived to occur more easily.

#### 3.1.3 Weak professional responsibility and attitudes toward care

Some participants described challenges in maintaining consistent attention to routine nursing care, particularly under conditions of workload pressure, fatigue, and repetitive clinical tasks. Participants reported that some nursing activities perceived as less urgent were occasionally delayed or unintentionally overlooked.

One participant described postponing routine care because it was not perceived as immediately necessary:

“At that time, I thought oral care was not urgent, so I postponed it, and later I simply forgot about it.” (C6)

Another participant reflected on reduced vigilance during routine patient assessment:

“I did not conduct rounds as required and developed a sense of complacency. I thought the patient's condition was stable and did not need such careful assessment, so I skipped several evaluation procedures. As a result, I almost failed to detect subtle changes in the patient's condition.” (C12)

Participants also described emotional fatigue associated with repetitive nursing work, which sometimes reduced their motivation to consistently perform routine nursing tasks.

“Facing repetitive basic nursing tasks every day gradually makes me feel burned out. My motivation to actively carry out nursing procedures decreases, and I unconsciously skip some tasks that seem unimportant, which eventually results in missed nursing care.” (C7)

Participants suggested that, during the early stage of professional development, some junior nurses were still developing awareness of the importance of fundamental nursing care and adapting to professional responsibilities. Under demanding clinical conditions, routine or non-urgent nursing activities were sometimes de-prioritized, increasing the possibility of missed nursing care.

### 3.2 Theme 2—organizational factors influencing missed nursing care

This theme demonstrates how organizational-level factors influenced the occurrence of missed nursing care among junior nurses. Participants described that inadequate staffing, excessive workload, insufficient supervisory and professional support, and limited opportunities for career development shaped their ability to provide comprehensive nursing care.

#### 3.2.1 Inadequate staffing and imbalanced workload

Participants frequently identified inadequate staffing and an excessive workload as important organizational factors contributing to missed nursing care. Heavy patient loads, particularly during night shifts and emergency situations, were described as creating competing demands that made it difficult to complete all required nursing activities.

One participant described the burden of caring for critically ill patients while managing multiple nursing responsibilities:

“Taking care of one critically ill patient is already exhausting, yet I am still expected to complete all nursing procedures.” (C8)

Another participant highlighted the challenges of managing simultaneous admissions during night shifts:

“During night shifts, several critically ill patients may arrive at the same time, and I simply cannot manage to complete everything properly.” (C7)

Participants commonly reported that insufficient staffing and time pressure limited their ability to provide comprehensive nursing care. Under demanding clinical conditions, urgent treatment-related tasks were often prioritized, while some routine nursing activities, psychological support, and health education were delayed or overlooked.

#### 3.2.2 Deficiencies in clinical practice standards and supervisory support

Participants described unclear clinical operational standards and insufficient supervisory support as organizational factors contributing to missed nursing care. Uncertainty regarding clinical procedures, limited access to timely consultation, and insufficient professional guidance were commonly reported.

One participant described uncertainty in clinical procedures and limited access to practical training and consultation:

“I was not very certain about the appropriate frequency of wound dressing changes and could only rely on experience. Once, this almost led to an infection. Our department lacks systematic advanced training for clinical practical skills, and when I encounter unclear procedural standards, there is no convenient or authoritative channel for consultation, so I can only figure things out on my own.” (C10)

Another participant emphasized difficulties in improving clinical practice in the absence of timely feedback and mentorship:

“After documentation errors occurred, I did not receive effective feedback and did not know how to improve. When I encountered nursing practice issues that were difficult to judge in daily work, timely professional guidance was often unavailable. The hospital has not established a fixed one-to-one mentorship or consultation mechanism, so improving professional competence largely depends on figuring things out independently while working.” (C11)

Participants suggested that the absence of clear procedural guidance, timely feedback, and structured mentorship made it difficult for junior nurses to manage uncertain clinical situations. Newly employed nurses, in particular, reported challenges in making clinical judgments when accessible professional support was unavailable, which sometimes contributed to delayed or incomplete nursing care.

#### 3.2.3 Limited career advancement opportunities and inadequate incentives

##### 3.2.3.1 Mechanisms

Participants described limited career development opportunities and insufficient institutional incentives as factors influencing work motivation and professional engagement. Several participants perceived that opportunities for promotion and professional recognition were limited, particularly for nurses primarily engaged in frontline clinical work.

One participant described frustration regarding promotion opportunities and perceived an imbalance in professional evaluation:

“There are very few opportunities for promotion, and research achievements seem to be valued much more. This puts clinical nurses at a disadvantage. Many times, the effort spent managing clinical problems during demanding shifts does not compared to the resources gained from publishing a few papers. Over time, I gradually lost motivation to invest extra effort in improving clinical nursing practice.” (C12)

Another participant emphasized concerns regarding the relationship between effort and professional recognition:

“My effort does not feel proportional to the rewards I receive, and gradually I lost motivation. In our hospital, research output carries too much weight in salary evaluations and professional promotion systems, while indicators reflecting the value of frontline clinical work are undervalued. This further reduced my motivation to engage fully in nursing work and may eventually contribute to missed nursing care.” (C9)

Participants also reported limited access to structured professional development opportunities. Routine training was often described as focusing primarily on basic operational procedures, with fewer opportunities for specialty nursing development, leadership training, or individualized career guidance. Some participants further noted insufficient institutional support and protected time for continuing education or specialty training, which they perceived as affecting long-term professional engagement and clinical development.

### 3.3 Theme 3—external environmental influences

This theme demonstrates how external environmental factors influenced missed nursing care among junior nurses. Participants described that healthcare system transformation, evolving clinical demands, and increasing expectations from patients and family members created additional pressures that shaped nursing care delivery.

#### 3.3.1 Challenges arising from healthcare system transformation

Participants described rapid technological changes and evolving models of care as creating additional challenges for clinical adaptation. Difficulties in adapting to electronic systems, multidisciplinary collaboration, and changing clinical expectations were commonly reported.

One participant described challenges in using electronic medical record systems:

“I am not very proficient with the electronic medical record system, so it is easy for me to enter incorrect data or overlook important information.” (C12)

Another participant emphasized difficulties in adapting to multidisciplinary collaboration:

“Multidisciplinary team discussions involve knowledge from several different fields, and I often feel unable to cope with them.” (C10)

Participants suggested that increasing clinical and technological demands sometimes made it difficult for junior nurses to balance multiple responsibilities simultaneously. Under such circumstances, routine nursing activities requiring additional time and communication, such as health education and psychological support, were occasionally delayed or overlooked.

In addition, some participants described increasing expectations for nursing care associated with evolving healthcare service models. However, limited adjustments in staffing and workflow processes were perceived as making adaptation more challenging forjunior nurses.

#### 3.3.2 Expectations of patients and family members

Participants described expectations from patients and family members as an additional source of pressure during clinical practice. Concerns regarding complaints, misunderstandings, and difficulties in communication were commonly reported, particularly when junior nurses felt unable to fully meet patients' or caregivers' expectations.

One participant described anxiety related to communication with family members and concerns about complaints:

“If I cannot explain the patient's condition clearly, family members may complain about me or say that I have a poor attitude. Even minor nursing mistakes may lead to disputes, and I am constantly worried about not meeting their expectations. Staying in such a state of tension further increases my psychological burden at work.” (C2)

Another participant emphasized challenges related to family expectations and trust in professional judgment:

“Sometimes family members expect us to follow their preferences completely and do not trust our professional judgment. If we insist on following standardized procedures, conflicts can easily arise. This feeling of being distrustful makes me anxious every time I communicate with family members because I worry that saying the wrong thing may trigger conflict. Our daily work is already demanding, and constantly guarding against these situations is physically and emotionally exhausting.” (C8)

Participants reported that responding to repeated concerns, explanations, and emotional reassurance for patients and family members sometimes required considerable time and attention. Under such circumstances, junior nurses described difficulties in balancing communication demands with routine nursing responsibilities, and some nursing activities were occasionally delayed or overlooked.

## 4 Discussion

### 4.1 Individual-level factors contributing to missed nursing care among junior nurses

This study identified insufficient knowledge and skills, psychological stress during professional transition, and challenges related to professional responsibility as important individual-level factors associated with missed nursing care among junior nurses. These findings suggest that missed nursing care among junior nurses may reflect not only limitations in clinical competence but also the challenges of adapting to complex clinical responsibilities during the early stages of professional practice.

Participants frequently described difficulties with clinical judgment, prioritization, and emergency response, particularly when caring for patients with complex or rapidly changing conditions. Consistent with previous studies, limited clinical experience and insufficient competence may reduce confidence in decision-making and contribute to uncertainty when responding to clinical changes, which may partially explain delayed or incomplete nursing care among junior nurses (24–26). Newly employed nurses are often required to manage multiple competing clinical demands while simultaneously adapting to unfamiliar responsibilities, making task prioritization particularly challenging in high-pressure environments.

Psychological burden and role transition also emerged as important findings in this study. Participants described persistent stress, fatigue, and uncertainty during the transition from student to registered nurse. Similar findings have been reported in studies of professional transition, which suggest that transition shock, emotional exhaustion, and limited coping experience may influence nurses' clinical performance and confidence (27, 28). Under sustained psychological strain, junior nurses may experience difficulties maintaining concentration and managing competing responsibilities, which may contribute to omissions in routine nursing care. These findings highlight the importance of strengthening transition support and psychological resilience among newly employed nurses (23, 29, 30). Structured mentorship, simulation-based training, peer support, and stress-management interventions may help facilitate adaptation and improve care consistency (31).

Furthermore, some participants described reduced motivation toward routine nursing care and difficulties maintaining vigilance in repetitive clinical tasks, particularly when care activities were perceived as non-urgent. Existing literature suggests that professional identity and occupational commitment are closely associated with nursing quality and professional engagement (32, 33). Repetitive workloads, emotional fatigue, and insufficient recognition may weaken motivation toward fundamental nursing care (34). Therefore, reflective supervision, supportive management, and opportunities for professional development may help strengthen professional responsibility and support care quality among junior nurses.

### 4.2 Organizational-level barriers and implications for nursing management

At the organizational level, inadequate staffing, excessive workload, insufficient supervisory support, and limited career development opportunities emerged as important factors associated with missed nursing care among junior nurses (35, 36). These findings suggest that missed nursing care may reflect broader organizational and managerial challenges rather than solely individual performance issues.

Consistent with previous research, participants described staffing shortages and high work loads as important contextual pressures, particularly during night shifts and emergency situations (37, 38). Under conditions of limited time and competing clinical priorities, junior nurses often reported difficulties balancing urgent technical procedures with routine nursing care, psychological support, and health education. These findings may help explain why some aspects of nursing care are more likely to be delayed or overlooked in resource-constrained clinical environments. Strengthening workforce allocation and improving workload distribution may therefore support more consistent nursing care delivery (8, 34).

Participants also highlighted challenges related to inadequate training, insufficient feedback, and limited supervisory support. Similar findings have been reported in previous studies, suggesting that the absence of structured mentorship and accessible professional guidance may contribute to uncertainty in clinical decision-making and variability in nursing practice (39). Newly employed nurses, in particular, may experience greater difficulties managing complex or ambiguous clinical situations when timely support is unavailable. These findings underscore the importance of structured onboarding programs, tiered clinical training, mentorship, and ongoing professional feedback to facilitate professional adaptation and strengthen nursing consistency.

In addition, participants described limited opportunities for career development and concerns regarding professional recognition. Previous evidence suggests that insufficient organizational support and perceived inequity in recognition may influence professional engagement, job satisfaction, and retention among nurses (40). Supporting transparent career pathways, continuing education, and opportunities for specialty development may help sustain professional motivation and strengthen long-term workforce stability (41).

### 4.3 External environmental pressures and system-level challenges

This study further suggests that healthcare system transformation and expectations from patients and family members represent important external influences associated with missed nursing care amongjunior nurses.

Participants described rapid technological changes and evolving care models as creating challenges for clinical adaptation. Difficulties in adapting to electronic medical record systems, multidisciplinary collaboration, and expanding expectations for comprehensive nursing care were commonly reported. Previous studies similarly suggest that technological transitions and increasing clinical complexity may influence nurses’ workload and clinical adaptation (40). Under competing demands, junior nurses may experience difficulties balancing documentation requirements, physician orders, and nursing activities requiring greater communication and interpersonal engagement, such as health education and emotional support. These Findings highlight the potential importance of digital competency training and interdisciplinary collaboration support in contemporary nursing practice.

In addition, participants described expectations from patients and family members as a source of emotional pressure, particularly in situations involving concerns about complaints, misunderstandings, and communication difficulties. Similar findings have shown that concerns regarding conflict and dissatisfaction may contribute to emotional burden and communication stress among nurses (42–44). Junior nurses may spend substantial time responding to repeated questions, explanations, and reassurance, which may affect the time available for routine nursing care. Strengthening communication training and patient—family education may therefore support more effective nurse—patient relationships and reduce communication-related burdens in clinical practice.

Taken together, these findings suggest that missed nursing care among junior nurses may be influenced by the interaction of individual, organizational, and external contextual factors. Addressing missed nursing care may therefore require integrated strategies that support clinical competence, organizational adaptation, and effective communication in complex healthcare environments.

## 5 Limitations

Several limitations should be acknowledged. First, participants were recruited from a single tertiary hospital in Shanghai, and the relatively small sample may limit the transferability of the findings to other healthcare settings and nursing populations. Second, this study focused exclusively on junior nurses; therefore, the experiences and perspectives of nurses at other career stages were not captured. Third, the study explored missed nursing care solely from the perspective of junior nurses without incorporating the views of patients or family members, which may limit a comprehensive understanding of its broader impact. Despite these limitations, the findings provide valuable insights into the factors influencing missed nursing care among junior nurses and offer directions for future research. Future studies should involve multiple healthcare settings, larger and more diverse samples, and include additional stakeholder perspectives to further validate and extend these findings.

## 6 Conclusions

This study revealed that missed nursing care among junior nurses is shaped by the interaction of individual, organizational, and external environmental factors. The findings highlight the complexity of missed nursing care and underscore the challenges faced by junior nurses during their transition into professional practice. Addressing missed nursing care requires comprehensive strategies that strengthen clinical competence, psychological support, staffing management, and organizational support systems. By providing an in-depth understanding of junior nurses' experiences, this study offers evidence to inform targeted interventions aimed at improving nursing care quality and patient safety. Future research involving multiple healthcare settings and broader stakeholder perspectives is warranted to further validate and expand these findings.

## Data Availability

The original contributions presented in the study are included in the article/Supplementary Material, further inquiries can be directed to the corresponding author/s.
