# Supplementary material for: Perceptions and experiences of missed nursing care among junior nurses: a qualitative study using reflexive thematic analysis
Source: Front Health Serv. 2026 Jul 20;6:1853426. doi: 10.3389/frhs.2026.1853426 (PMC13429587; doi:10.3389/frhs.2026.1853426)
Supplement: Supplementary file 1 [file Supplementaryfile1.doc]

**Supplementary Material 1**

**Informed Consent Form**

Dear Nurse, Hello!

We sincerely invite you to participate in this study. Before deciding whether to participate, please carefully read the following information. If you have any questions, please feel free to ask the research team at any time. Your participation is entirely voluntary. You have the right to refuse or withdraw from this study at any time without any negative consequences.

**1. Purpose of the Study**

This study aims to gain an in-depth understanding of junior nurses' (registered nurses with ≤3 years of clinical experience) real perceptions and lived experiences of missed nursing care in clinical practice. It explores junior nurses' psychological feelings, contributing factors, and coping strategies when facing missed nursing care, with the goal of providing evidence for improving nursing quality, optimizing nursing workforce allocation, and developing targeted support measures.

**2. Research Methods**

This study adopts a qualitative phenomenological approach, using semi-structured in-depth interviews to collect data. If you agree to participate, we will arrange a time and location (e.g. a private and comfortable room in the hospital conference room) for a face-to-face in-depth interview, each lasting approximately 30–60 minutes. With your consent, the interview will be audio-recorded to ensure accurate transcription and analysis. The recordings will be used solely for this study and will be securely stored after the interview.

**3. Possible Risks and Discomfort**

This study involves recalling and reflecting on your experiences of missed nursing care in clinical practice, which may evoke stressful, frustrating, or unpleasant memories and cause a certain degree of emotional discomfort.

If you feel any discomfort during the interview, you may request to pause or terminate the interview at any time. The research team will fully respect your

decision.

This study does not involve any invasive procedures or physical harm; the overall risk is minimal.

**4. Possible Benefits**

Participation may not provide direct personal benefits. However, your sharing will contribute to a better understanding of the challenges faced by junior nurses, promote improvements in nursing management policies, and ultimately benefit nursing professionals—including yourself. If you need psychological support after the interview, we can provide relevant mental health resource information.

**5. Confidentiality and Privacy Protection**

**Anonymity**: This study uses an anonymous approach. Your real name will not appear in any materials; you will be identified by a code (e.g., C1, C2, …).

**Information Confidentiality:** All personal information and interview content you provide will be kept strictly confidential and accessible only to members of the research team.

**Data Storage:** Audio recordings and transcribed texts will be stored on encrypted computers/mobile devices with password protection, accessible only to the researchers. After the study concludes, raw data will be retained for 5 years and then destroyed.

**Publication:** When citing your data in research papers or academic reports, no personally identifiable information will be disclosed.

**Exceptions to Confidentiality:** If you disclose during the interview any information indicating an imminent risk of serious harm to yourself or others (e.g., self-harm, harm to others), the researchers are obligated to report to the relevant authorities in accordance with applicable laws, regulations, and ethical requirements. In such cases, only the minimum necessary information will be disclosed.

**6. Voluntary Participation and Right to Withdraw**

Your participation is entirely voluntary. Declining to participate or withdrawing midway will not have any adverse effects on your work assessment, promotion, salary, or any other employment-related matters.

You have the right to withdraw unconditionally at any stage of the study without providing a reason. Upon withdrawal, you may request deletion of data already provided (data can be unconditionally deleted before the interview begins; if the interview is in progress or has been completed, data may not be individually removable from the analysis due to de-identification, but we will cease using any new data from you).

**7. Researcher Information**

I have read (or had the content read aloud and explained to me by the researcher) the entire informed consent form above. I fully understand the purpose, methods, possible risks, benefits, confidentiality measures, and my rights. I voluntarily agree to participate in this study.

Signature Date

Participant

Researcher

**Supplementary Material 2**

**Structured interview guide for Perceptions and Experiences of Missed Nursing Care Among Junior Nurses**

1、 —Could you share your experience of missed nursing care during your time as a junior nurse?

— Can you describe a specific incident that comes to mind?

2. — How do you perceive omissions of nursing care?

— What do you think are the various causes contributing to missed nursing care?

— What do you think is the impact of missed nursing care on patients?

3. —What were your feelings or reflections when facing such an event?

4. How do you perceive the impact of missed nursing care on your overall

education and professional development?

5、—What kind of support do you think is needed to address missed nursing care?

6,—Is there any additional information you would like to share that we have not

covered?

Note: The interview guide was designed to be participant-centred, allowing flexibility for follow-up questions and in-depth exploration of responses .

**Supplementary Material 3: Ethical Approval Statement**


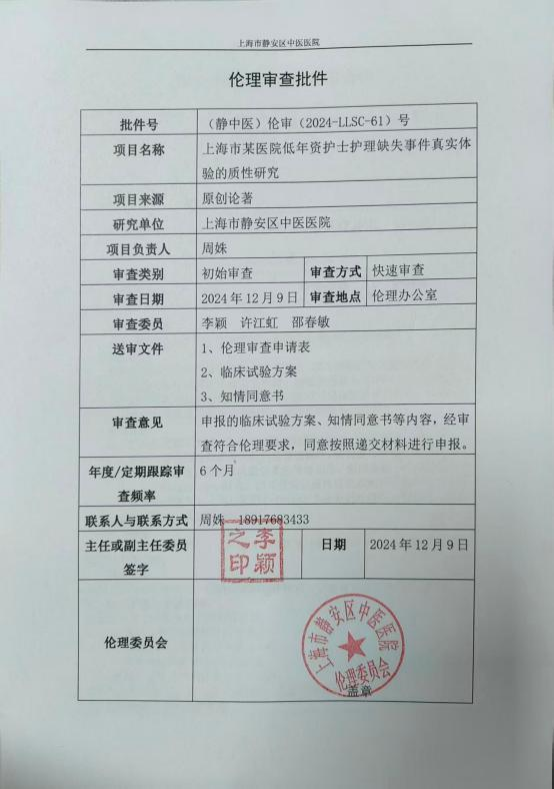


**Supplementary Material 4:**

**Table 1. General characteristics of the participants**

| **Number** | **Age** | **Gender** | **Education** | **Department** |
| --- | --- | --- | --- | --- |
| **C1** | 23 | Female | Bachelor | Internal Medicine |
| **C2** | 22 | Female | Bachelor | Internal Medicine |
| **C3** | 24 | Female | Bachelor | Surgery |
| **C4** | 23 | Female | Junior | Surgery |
| **C5** | 21 | Female | Junior | Internal Medicine |
| **C6** | 22 | Female | Bachelor | ICU |
| **C7** | 25 | Female | Master | ICU |
| **C8** | 24 | Male | Bachelor | Surgery |
| **C9** | 23 | Female | Junior | Internal Medicine |
| **C10** | 24 | Female | Junior | Surgery |
| **C11** | 23 | Female | Bachelor | Internal Medicine |
| **C12** | 24 | Male | Bachelor | Internal Medicine |

**Supplementary Material 5:**

**Table 2. Analytical Process of Reflexive Thematic Analysis**

| ****Phase**** | ****Description**** | ****Outcome**** |
| --- | --- | --- |
| Familiarization with the data | Interview recordings were transcribed verbatim. Researchers repeatedly read the transcripts and recorded initial reflections. | Familiarity with the dataset |
| Generating initial codes | Meaningful data segments were systematically coded across all transcripts. | Initial code set |
| Constructing candidate themes | Similar codes were grouped into potential themes and subthemes. | Preliminary thematic structure |
| Reviewing and refining themes | Themes were checked against the coded extracts and the entire dataset, then revised where necessary. | Refined themes |
| Defining and naming themes | The essence, boundaries, and names of each theme were finalized. | Clearly defined themes |
| Producing the final thematic framework | Themes were finalized and supported by representative quotations, resulting in the final thematic framework. | **Three themes and ten subthemes** |

****Supplementary Material 6: Table 3. Coding Framework of the Reflexive Thematic Analysis****

| ****Representative Data Extract / Meaning**** | ****Initial Code**** | ****Subtheme**** | ****Theme**** |
| --- | --- | --- | --- |
| **"I often lacked confidence when dealing with complex clinical situations."** | **Lack of confidence** | **Insufficient professional knowledge and skills** | **Individual-level factors** |
| **"I was unfamiliar with some nursing procedures."** | **Insufficient clinical competence** | **Insufficient professional knowledge and skills** | **Individual-level factors** |
| **"I was afraid of making mistakes."** | **Fear of errors** | **Psychological stress and professional adaptation** | **Individual-level factors** |
| **"The transition from student to nurse was overwhelming."** | **Difficulty adapting to the professional role** | **Psychological stress and professional adaptation** | **Individual-level factors** |
| **"Sometimes I overlooked tasks because I was too busy."** | **Poor task prioritization** | **Weak responsibility and work attitude** | **Individual-level factors** |
| **"There were not enough nurses on duty."** | **Staff shortage** | **Human resource constraints** | **Organizational management factors** |
| **"Workload was extremely heavy during peak shifts."** | **Heavy workload** | **Human resource constraints** | **Organizational management factors** |
| **"Senior nurses had little time to supervise us."** | **Inadequate supervision** | **Deficiencies in quality management and supervision** | **Organizational management factors** |
| **"Training focused more on theory than practice."** | **Insufficient practical training** | **Inadequate training and professional development** | **Organizational management factors** |
| **"Communication between nurses and physicians was inefficient."** | **Poor interdisciplinary communication** | **Inefficient communication and teamwork** | **Organizational management factors** |
| **"Patients expected immediate responses to every request."** | **High patient expectations** | **Increasing patient and family expectations** | **External environmental factors** |
| **"Electronic documentation took a lot of time."** | **Documentation burden** | **Healthcare system transformation** | **External environmental factors** |
| **"Family members often questioned our competence."** | **Family pressure** | **Social and occupational pressures** | **External environmental factors** |

**Supplementary Material 7. Consolidated criteria for reporting qualitative studies (COREQ): 32-item checklist**

| **No.** | **Item** | | **Guide questions / description** | |
| --- | --- | --- | --- | --- |
|  | |  | | |
| 1. | Interviewer /  facilitator | | Which author/s conducted the interviews or focus group? | Xiaojuan Liu |
| 2. | Credentials | | What were the researcher’s credentials? E.g., PhD, MD | Rongrong Zhou, Xiaojuan Liu, Shu Zhou = MA Bihua Shen, Shuirong Zhan = BA  Yanjun Mao = PhD |
| 3. | Occupation | | What was their occupation at the time of the study? | All are medical personnel. |
| 4. | Gender | | Was the researcher male or female? | All are females |
| 5. | Experience and training | | What experience or training did the researcher have? | Yanjun Mao received training in qualitative research methods during her PhD studies. Rongrong Zhou, Xiaojuan Liu, Shu Zhou an experienced qualitative researcher with multiple peer-reviewed publications using qualitative methodologies in health systems research. Bihua Shen and Shuirong Zhan have no formal qualitative training; they devote themselves to clinical teaching and management; they possess abundant clinical expertise. |
|  | |  | | |
| 6. | Relationship  established | | Was a relationship established prior to study commencement? | In most cases, no prior relationship existed between the interviewer and participants. A professional relationship existed only with some interviewees from the Ministry of Health. |
| 7. | Participant  knowledge of the interviewer | | What did the participants know about the researcher? E.g., personal goals, reasons for doing the research | They knew the interviewer was part of the study team conducting research on Perceptions and Experiences of Missed Nursing Care |

| 8. | Interviewer  characteristics | | What characteristics were reported about the interviewer/facilitator? E.g., bias, assumptions, reasons and interests in the research topic | Interviewees were informed in advance, via email, about her role and the study objectives. Given thieirs professional background, they were aware of potential assumptions and interests in the topic and therefore took care to maintain a neutral and open approach during the interviews. |
| --- | --- | --- | --- | --- |
|  | |  | | |
| 9. | Methodological orientation and theory | | What methodological orientation was stated to underpin the study? E.g., grounded theory, discourse analysis, phenomenology, content analysis | The study was underpinned by a Qualitative Study study based on data generated through semi-structured interviews. The analysis combined deductive codes derived from the interview guide with inductive codes that emerged from the data. |
| 10. | Sampling | | How were participants selected? E.g., purposive, convenience, consecutive, snowball | purposive |
| 11. | Method of  approach | | How were participants approached? E.g. face-to-face, telephone, mail, email | Face-to-face. |
| 12. | Sample size | | How many participants were in the study? | 12 interviews were conducted. |
| 13. | Non-participation | | How many people refused to participate or dropped out? Reasons? | There were no participants who dropped out of the study. |
| 14. | Setting of data collection | | Where was the data collected? E.g., home, clinic, workplace | Face-to-face (12 workplaces) |
| 15. | Presence of  non-participants | | Was anyone else present besides the participants and researchers? | Only participants and researcher. |

| 16. | Description of the sample | What are the important characteristics of the sample? E.g., demographic data, date | 12 participants: **Gender:** 10 women, 2 men (Table 1). Department: 6 Internal Medicine,4 Surgery,2 ICU**.Education**:4 Bachelor,7Junior, Master |
| --- | --- | --- | --- |
| 17 | Interview guide | Were questions, prompts, guides provided by the authors? Was it pilot tested? | Semi-structured interview guide |
| 18. | Repeat  interviews | Were repeat interviews carried out?  If yes, how many? | No. |
| 19. | Audio / visual recording | Did the research use audio or visual recording to collect the data? | Yes. face-to-face and telephone interviews were audio-recorded only. |
| 20. | Field notes | Were field notes made during and/or after the interviews? | Yes |
| 21. | Duration | What was the duration of the interviews of focus groups? | Interviews lasted 30–60 minutes. |
| 22. | Data saturation | Was data saturation discussed? | Yes |
| 23. | Transcripts returned | Were transcripts returned to participants for comments and/or corrections? | No |
| **Domain 3: Analysis and Findings *Data analysis*** | | | |
| 24. | Number of data coders | How many data coders coded the data? | Two |

| 25. | Description of the coding tree | Did authors provide a description of the coding tree? | No |
| --- | --- | --- | --- |
| 26. | Derivation of themes | Were themes identified in advance or derived from the data? | Themes were identified through a combination of deductive coding based on the interview guide and inductive coding derived from the data. |
| 27. | Software | What software, if applicable, was used to manage the data? | ATLAS.ti software was used to manage, code, and analyze the interview transcripts. |
| 28. | Participant checking | Did participants provide feedback on the findings? | No |
| ***Reporting*** | |  | |
| 29. | Quotations  presented | Were participant quotations presented to illustrate the themes / findings?  Was each quotation identified? E.g., participant number | Yes  Yes |
| 30. | Data and findings consistent | Was there consistency between the data presented and the findings? | Yes |
| 31. | Clarity of major themes | Were major themes clearly presented in the findings? | 3 key themes were identified in the study and presented in the manuscript. All of the themes were discussed in the results section. |
| 32. | Clarity of minor themes | Is there a description of diverse cases or discussion of minor themes? | Yes. Diverse cases were presented to reflect differences across stakeholder groups. |

Reference:

Tong, A., Sainsbury, P., Craig, J. (2007) Consolidated criteria for reporting qualitative research (COREQ): a 32-item checklist for interviews and focus groups. International Journal Quality Health Care 19(6):349–357
